# Poliovirus receptor (PVR)-like protein cosignaling network: new opportunities for cancer immunotherapy

**DOI:** 10.1186/s13046-021-02068-5

**Published:** 2021-08-25

**Authors:** Baokang Wu, Chongli Zhong, Qi Lang, Zhiyun Liang, Yizhou Zhang, Xin Zhao, Yang Yu, Heming Zhang, Feng Xu, Yu Tian

**Affiliations:** 1 Department of General Surgery, Shengjing Hospital of China Medical University, Shenyang, 110004 Liaoning Province China; 2 Department of Surgery, Jinzhou Medical University, Jinzhou, 121001 Liaoning Province China; 3 Department of College of Medical and Biological Information Engineering, Northeastern University, Shenyang, 110819 Liaoning Province China

**Keywords:** PVR, Cosignaling network, Receptor, Ligand, Cancer immunotherapy

## Abstract

Immune checkpoint molecules, also known as cosignaling molecules, are pivotal cell-surface molecules that control immune cell responses by either promoting (costimulatory molecules) or inhibiting (coinhibitory molecules) a signal. These molecules have been studied for many years. The application of immune checkpoint drugs in the clinic provides hope for cancer patients. Recently, the poliovirus receptor (PVR)-like protein cosignaling network, which involves several immune checkpoint receptors, i.e., DNAM-1 (DNAX accessory molecule-1, CD226), TIGIT (T-cell immunoglobulin (Ig) and immunoreceptor tyrosine-based inhibitory motif (ITIM)), CD96 (T cell activation, increased late expression (TACLILE)), and CD112R (PVRIG), which interact with their ligands CD155 (PVR/Necl-5), CD112 (PVRL2/nectin-2), CD111 (PVRL1/nectin-1), CD113 (PVRL3/nectin-3), and Nectin4, was discovered. As important components of the immune system, natural killer (NK) and T cells play a vital role in eliminating and killing foreign pathogens and abnormal cells in the body. Recently, increasing evidence has suggested that this novel cosignaling network axis costimulates and coinhibits NK and T cell activation to eliminate cancer cells after engaging with ligands, and this activity may be effectively targeted for cancer immunotherapy. In this article, we review recent advances in research on this novel cosignaling network. We also briefly outline the structure of this cosignaling network, the signaling cascades and mechanisms involved after receptors engage with ligands, and how this novel cosignaling network costimulates and coinhibits NK cell and T cell activation for cancer immunotherapy. Additionally, this review comprehensively summarizes the application of this new network in preclinical trials and clinical trials. This review provides a new immunotherapeutic strategy for cancer treatment.

## Background

Immune checkpoint molecules, also known as cosignaling molecules, are pivotal cell-surface molecules controlling immune cell responses that either stimulate a signal (costimulatory molecules) or inhibit a signal (coinhibitory molecules) [1]. Checkpoint molecules mainly comprise members of the immunoglobulin superfamily and tumor necrosis factor/receptor superfamily [2]. Costimulatory molecules enhance the immune response by promoting a signal, whereas coinhibitory molecules attenuate the immune response by inhibiting a signal [3]. Natural killer (NK) cells and T cells are important components of the immune system, and their surface checkpoint molecules have been intensively studied. To date, several signaling pathways mediated by immune checkpoint molecules in immune cells have been reported to modulate the immune response while protecting against and killing virus-infected cells or malignant neoplastic cells. CTLA-4, as an immune checkpoint molecule, has been intensively investigated since its discovery [3–7]. Subsequently, PD-1, another immune checkpoint molecule, inhibits T cell cytotoxicity after binding with its ligands PD-L1/PD-L2 [8]. However, the immune checkpoint molecules mentioned above are secondary signals in the immune system that involve costimulatory or coinhibitory T cell cytotoxicity but not NK cell cytotoxicity. Similar to T cells, stimulatory and inhibitory receptors exist on the NK cell surface and guarantee that NK cells efficiently kill invading pathogens and infected or transformed cells but not normal cells by exerting cytotoxic effects after binding to their ligands [9].

Currently, researchers have developed drugs targeting checkpoint molecules for the clinical treatment of cancer. Ipilimumab is a monoclonal antibody (mAb) that enhances T cell activity by specifically targeting cytotoxic T-lymphocyte-associated protein 4 (CTLA-4) and was the first drug to be used in the clinic to significantly prolong survival in patients with advanced melanoma [3, 10]. Additionally, after this mAb drug was developed, two additional mAb drugs, pembrolizumab and nivolumab, were developed to treat cancers by selectively inhibiting the programmed cell death protein 1 (PD-1)/programmed death-ligand 1 (PD-L1) inhibitory pathway. The cancer types that are responsive to PD-1 therapy with an objective clinical response include advanced melanoma, nonsmall-cell lung cancer, and kidney cancer [11]. The emergence of these mAbs is a milestone in cancer immunotherapy. However, clinical results indicate that many types of cancer, including pancreatic ductal adenocarcinoma (PDAC), colon cancer, prostate cancer, and ovarian cancer, do not respond or respond poorly to PD-1 blockade therapy [11].

Recently, poliovirus receptor (PVR)-like proteins, which are expressed on most immune cells, including NK cells and T cells, were identified. These proteins include DNAX accessory molecule-1 (DNAM-1, CD226); T-cell immunoglobulin (Ig) and immunoreceptor tyrosine-based inhibitory motif (ITIM) (TIGIT); T cell activation, increased late expression (CD96, also identified as TACLILE); and CD112R (PVRIG). PVR-like proteins belong to a newly emerging immunoglobulin superfamily of proteins containing a PVR signature motif in the Ig variable-like (IgV) domain. This group of proteins initially attracted attention as adhesion proteins that mediate epithelial cell-cell contacts. The two main ligands, CD155 (PVR/Necl-5) and CD112 (PVRL2/nectin-2), can interact with these four receptors. In addition, other ligands, such as CD111 (PVRL1/nectin-1), CD113 (PVRL3/nectin-3), and Nectin4 (PVRL4), also interact with these receptors. In this review, we describe a new cosignaling network comprising four receptors and their ligands. This novel cosignaling pathway critically modulates the immune cell response and acts as a valuable checkpoint for cancer immunotherapy by regulating NK cells and T cells. However, the molecular and functional relationships among the components of the cosignaling pathway are unclear and need to be further researched.

## PVR like protein receptors

### DNAM-1

DNAM1 is a 65-kD immunoglobulin-like transmembrane glycoprotein consisting of an extracellular region with two IgV-like domains, a transmembrane region, a cytoplasmic region with an Ig tail-tyrosine (ITT), and four putative tyrosine residues and one serine residue that are phosphorylated [12, 13]. DNAM-1 is mainly expressed on the majority of monocytes, T cells, NK cells and platelets and on small subsets of B cells [12]. DNAM-1 is a costimulatory receptor that promotes intercellular adhesion, lymphocyte signaling, and lymphokine secretion and enhances NK cell and CTL cytotoxicity [12]. CD112/nectin2 and CD155/PVR have been identified as ligands of the DNAM1 receptor [14, 15], and the interaction between these ligands and receptors ensures NK or T cell-mediated lysis of tumor cells.

Although some studies have investigated the cell-intrinsic signaling cascade, synergy with other NK receptors and cell-extrinsic mechanisms after DNAM-1 interacts with its ligands are described in the following section. However, further research is needed.

### Intrinsic signaling cascades and synergy with other NK receptors

**Fig. 1:**
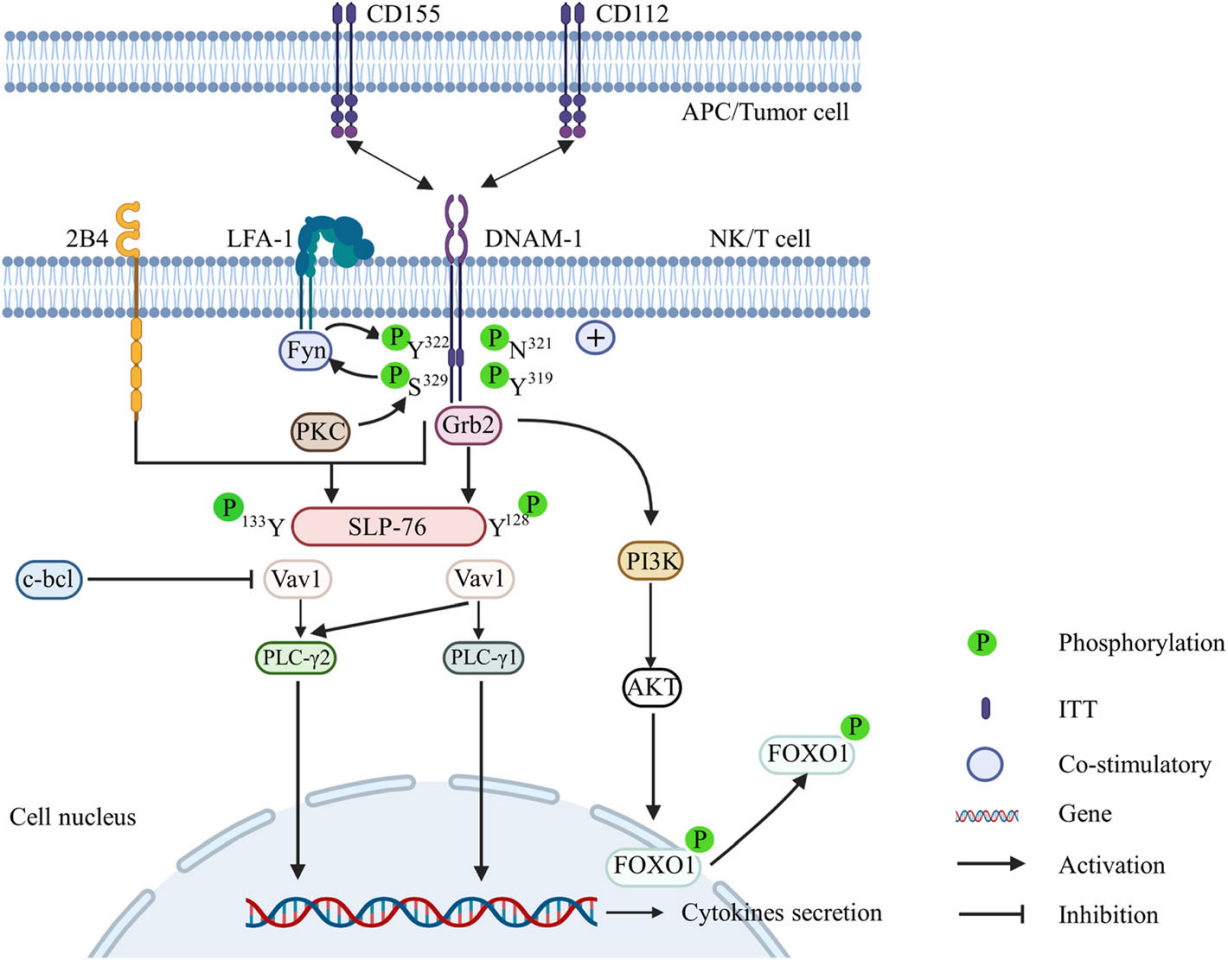
Created with BioRender.com. The bidirectional arrow represents the interaction between DNAM-1 and its ligands. DNAM-1 exerts a costimulatory effect after engagement with its ligands CD155 and CD112. The intrinsic cell signaling cascade of DNAM-1 subsequently occurs in NK cells, and the sites of DNAM-1 S^329^ and Y^322^ are phosphorylated under the synergistic action of PKC and Fyn src kinase induced by LFA-1, resulting in dual SLP-76 phosphorylation at Y^128^ and Y^113^. This phosphorylated form of SLP-76 binds to Vav1 molecules to activate PLC-γ2. Additionally, phosphorylated DNAM-1 recruits the adaptor Grb2, which leads to the activation of Vav-1, PI3K, and phospholipase C-γ1 (PLC-γ1). Both pathways lead to NK cell cytotoxicity changes

In NK cells, after DNAM-1 engages with its ligands CD112 and CD155 (Fig. 1), the Ser^329^ residue of DNAM-1 is phosphorylated by protein kinase C (PKC). Phosphorylated DNAM-1 subsequently crosslinks with leucocyte function-associated antigen-1 (LFA-1), resulting in lipid raft relocation and engagement with the cytoskeleton due to cooperation with the MAGUK homolog, large human discs, and the actin-binding protein 4.1G [16–18]. Then, the phosphorylation of the Y^322^ residue of DNAM-1 by Fyn src kinase is induced by LFA-1 [19]. It is now clear that DNAM-1 alone is not sufficient to trigger NK activation. Studies have shown that DNAM-1 synergizes with 2B4 to costimulate NK cells by phosphorylating the Y^128^ and Y^113^ residues of SLP-76 (SH2 domain-containing leukocyte phosphoprotein of 76 kDa). The dual phosphorylated form of SLP-76 binds to two Vav1 molecules, which activates phospholipase Cγ2 (PLC-γ2), resulting in degranulation, cytokine secretion, and Ca^2+^ flux [20, 21]. Additionally, DNAM-1 synergizes with 2B4 to overcome the inhibition of Vav1 by the E3 ubiquitin ligase c-cbl [20]. Recently, a study showed that upon recognition of its ligand, the asparagine residue at position 321 (N^321^) of DNAM-1 cooperates with the phosphorylated Y^319^ residue to recruit adaptor growth factor receptor-bound protein 2 (Grb2), leading to the activation of Vav-1, phosphatidylinositol 3 kinase (PI3K), and phospholipase C-γ1 (PLC-γ1) [13]. Furthermore, DNAM-1 promotes ERK and AKT activation. Activated AKT phosphorylates the downstream transcription factor FOXO1, a negative regulator of NK cell homing, late-stage maturation, and effector functions [22], and phosphorylated FOXO1 translocates from the nucleus to the cytoplasm, where it regulates natural killer cell antitumor responses [23].

### Cell-extrinsic signaling cascades

**Fig. 2:**
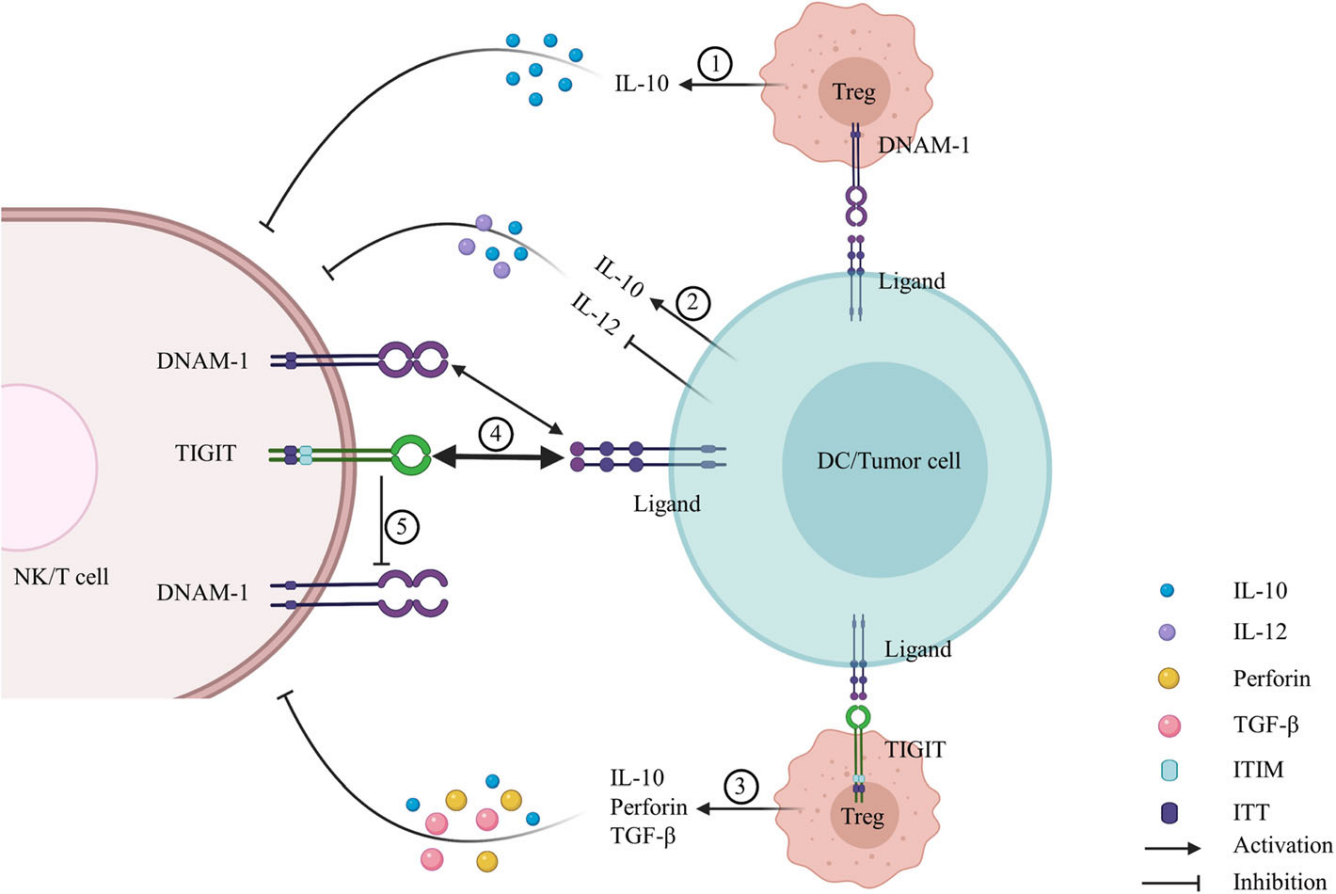
Created with BioRender.com. Receptors DNAM-1 and TIGIT cell extrinsic mechanisms: ① DNAM-1+ Tregs exert weak suppressive capacity with significantly increased cytokine IL-10 production. ② TIGIT binding to its ligands expressed on dendritic cells (DCs) inhibits T cell activation by enhancing the production of IL-10 and reducing the production of IL-12 in DCs, which creates an immunosuppressive microenvironment. ③ TIGIT+ Tregs exhibit enhanced suppressive capacity by augmenting Treg suppression and stability with high expression of IL-10, perforin, and TGF-β. ④ The bidirectional arrow represents the interaction between receptors and their ligands. The thickness of the arrow represents affinity between receptors and their common ligand. TIGIT indirectly inhibits T cell activation by directly completing DNAM-1 binding to their common ligand. ⑤ TIGIT inhibits T cell activation by disrupting CD226 cis-homodimerization in human T cells

In addition to being involved in intrinsic signaling cascades and synergizing with other NK receptors, DNAM-1 exerts a costimulatory effect by disrupting Tregs (Fig. 2), thus exerting an immunosuppressive effect on the microenvironment in humans. In melanoma, a low TIGIT/DNAM-1 ratio in Tregs can attenuate their suppressive function and stability, resulting in a decreased number of Tregs in tumors [24]. Additionally, DNAM-1+ TIGIT- Tregs are associated with a reduced Treg number and weak suppressive capacity after in vitro expansion while significantly increasing cytokine interleukin (IL)-10 production [25].

### Preclinical and clinical trials of DNAM-1-related strategies

Induction of the expression of DNAM-1 and its ligand represents a promising therapeutic strategy for cancer. An increasing number of studies have already reported that targeting DNAM-1 is a novel anticancer therapeutic strategy. Several preclinical (Table 1) and clinical trials (Table 2) of promising immune checkpoint-targeting strategies for cancer have been reported.

**Table 1:** Preclinical trials in promising cancer target of PVR like receptors

PVR like protein	Treatment	Immune cells	Tumor	Results	Reference
DNAM-1	Anti-DMAN-1 or anti-PVR mAbs	NK	Neuroblastoma	monoclonal antibody-mediated masking of either DNAM-1 (on NK cells) or PVR (on neuroblasts) resulted in strong inhibition of tumor cell lysis	[26]
DNAM-1	Anti-DMAN-1 or anti-PVR mAbs	NK	Tumor cell lines	The ability of NK-mediated lysis of tumor cells mediated by DNAM-1 engage with its ligands that was downregulated by mAb-mediated masking of the receptor or its ligands	[15]
DNAM-1	anti-CD226 mAb LeoA1	NK	Hepatoma	Crosslinking CD226 with the anti-CD226 mAb LeoA1 regulate miR-30c-1* expression, which promoted NK cell cytotoxicity against hepatoma cells by targeting HMBOX1	[27]
DNAM-1	DNAM-1 agonist	NK	Melanoma, experimental autoimmune encephalomyelitis	DNAM-1 agonist could activate DNAM-1 modifies the bidirectional crosstalk of NK cells with CD155 DC, which can suppress CNS autoimmunity and strengthen tumor surveillance	[28]
DNAM-1	Anti-CD226 mAb	Tregs	Allogeneic skin transplant	CD226 mAb promoted Treg expansion, reduced inflammation and prolonged allogeneic graft survival	[29]
DNAM-1	Anti-CD226 mAb	γδT	Hepatocellular carcinoma	Anti-DNAM-1 mAb-mediated masking experiments that γδT cells cytotoxicity against HCC cells as well as IFN-γ production were decreased	[30]
DNAM-1	CD226 agonist antibody	CD8+ T	pancreatic ductal adenocarcinoma	CD226 agonist antibody-mediated activation of CD226 augments the effect of TIGIT or PD-1 blockade on CD8 T-cell responses	[31]
TIGIT	Anti-TIGIT	NK	Colon cancer	Blockade of TIGIT prevented NK cell exhaustion and promoted NK cell–dependent tumor immunity, enhanced therapy with antibody to the PD-1 ligand PD-L1	[32]
TIGIT	Anti-TIGIT	NK	Ovarian cancer	Blockade of TIGIT enhanced degranulation and interferon gamma (IFN-γ) production of NK cells in response to OC tumor cells	[33]
TIGIT	Anti-TIGIT	CD8+ T	Melanoma	TIGIT and PD-1 blockade should be further explored to elicit potent antitumor CD8+ T cell responses	[34]
TIGIT	Anti-TIGIT	CD8+ T	Gastric cancer	Blockade TIGIT enhanced CD8 T cell activation and improved survival in tumor bearing mice	[35]
TIGIT	Anti-TIGIT	CD8+ T	Multiple myeloma	Blockade TIGIT by mAb increased the effector function of MM patient CD8+ T cells and suppressed MM development	[36]
TIGIT	Anti-TIGIT	CD8+ T	Myeloma	Immune checkpoint blockade using mAb against TIGIT significantly restored CD8+ T exhaustion and prolonged myeloma control after stem cell transplantation	[37]
TIGIT	Anti-TIGIT	CD8+ T, Tregs	Head and neck squamous cell carcinoma	Anti-TIGIT treatment significantly reverse T-cell exhaustion and reduce the population of Tregs in vitro and in vivo	[38]
TIGIT	Anti-TIGIT	CD4+ T, CD8+ T, Tregs	Multiple myeloma	Anti-TIGIT mAb depleted FoxP3+ Tregs, increased proliferation of IFN-γ-producing CD4+ T cells, and overcame the inhibition effect of CD8+ T cell signaling and cell proliferation by PVR ligation	[39]
TIGIT	Anti-TIGIT	αβT, γδT, Tregs	Hematologic malignancies	Anti-TIGIT mAbs could restore αβT-cell function, prevent CD155 mediated inhibition of γδ T cells, depletion of Tregs, and direct killing of tumor cells	[40]
TIGIT	Anti-TIGIT	Effector T, Tregs	Glioblastoma	TIGIT a checkpoint blockade increased effector T cell function and downregulation of suppressive Tregs and TIDCs to enhance antitumor immunity and survival in glioblastoma	[41]
TIGIT	Anti-TIGIT	CD4+ Tregs	Ovarian cancer	Anti-TIGIT treatment reduced the proportion of CD4+ Tregs	[42]
CD96	Anti-CD96	NK	Melanoma lung metastases	Anti-CD96 enhances the NK cell IFN-γ-dependent effector function, which significantly reduced experimental and spontaneous lung metastases	[43]
CD96	Anti-CD96	NK	Hepatocellular carcinoma	Anti-CD96 antibody of blocking CD96 and its ligand CD155 interaction, the human NK cell lines cytotoxicity was restored and enhanced	[44]
CD96	Anti-CD96	NK	Tumor metastases	CD96 targeted antibodies promote NK cell anti-tumor activity	[45]
CD96	Anti-CD96	CD8+ T	Anti-tumor	Ab blockade on CD8+ T cells could eliminate IFN-γ and/or TNF-α production, which associated with CD8+ T cell activation	[46]
CD96	Anti-CD96	CD8+ T	Melanoma	Anti-CD96 therapy is effective to enhance CD8+ T activity and limit tumor growth	[47]
CD96	Anti-CD96	Th19	Inflammatory diseases	Blockade of CD96 significantly restored the expansion and inflammatory properties of CD96^high^ Th9 cells	[48]
CD112R	Anti-CD112R	NK	Breast cancer	Blockage CD112R could improve trastuzumab therapy for breast cancer by enhancing NK cells activity	[49]
CD112R	Anti-CD112R	CD8+ T	Melanoma, pancreatic cancer	Blockade of PVRIG increased CD8+ T-cell function, an effect enhanced by combination with TIGIT or PD-1 blockade	[50]

*mAb* monoclonal antibody, *CNS* central nervous system, *HCC* hepatocellular carcinoma, *PD-1* programmed cell death 1, *PD-L1* programmed cell death-ligand 1, *TIDCs* tumor-infiltrating dendritic cells

**Table 2:** Clinical trials in promising cancer target of PVR like receptors

NCT Number	Target	Agent	Estimated Enrollment	Phase	Condition	Recruitment Statue	Estimated Study Completion Date
NCT04099277	DNAM-1	LY3435151	2	aI/b1	Solid Tumor, Triple-negative Breast Cancer, Gastric Adenocarcinoma, Head and Neck Squamous Cell Carcinoma, Cervical Carcinoma, High Grade Serous Ovarian Carcinoma, Undifferentiated Pleomorphic Sarcoma, Leiomyosarcoma	Terminated	May 5, 2020
NCT04656535	TIGIT	AB154	46	0/I	Glioblastoma	Not yet recruiting	Jul, 2023
NCT03628677	TIGIT	AB154	66	I	Solid Tumor, Unspecified, Adult	Recruiting	Nov 10, 2021
NCT04262856	TIGIT	AB154	150	II	Non Small Cell Lung Cancer, Nonsquamous Non Small Cell Lung Cancer, Squamous Non Small Cell Lung Cancer, Lung Cance	Recruiting	Jun 23, 2022
NCT04736173	TIGIT	AB154	625	III	Non Small Cell Lung Cancer, Nonsquamous Non Small Cell Lung Cancer, Squamous Non-small-cell Lung Cancer, Lung Cancer	Recruiting	Jun 30, 2026
NCT03945253	TIGIT	ASP8374	6	I	Advanced Solid Tumors	Completed	Jun 12, 2020
NCT03260322	TIGIT	ASP8374	169	I	Advanced Solid Tumors	Active, not recruiting	Mar, 2022
NCT04693234	TIGIT	BGB-A1217	167	II	Cervical Cancer	Not yet recruiting	Mar 31, 2023
NCT04732494	TIGIT	BGB-A1217	280	II	Esophageal Squamous Cell Carcinoma	Recruiting	Mar, 2024
NCT04047862	TIGIT	BGB-A1217	234	I	Locally Advanced, Metastatic Solid Tumors	Recruiting	Aug, 2023
NCT04746924	TIGIT	BGB-A1217	605	III	Non-small Cell Lung Cancer	Not yet recruiting	Mar, 2025
NCT04150965	TIGIT	BMS-986207	104	I/II	Multiple Myeloma, Relapsed Refractory Multiple Myeloma	Recruiting	Mar 30, 2022
NCT04354246	TIGIT	COM902	45	I	Advanced Cancer, Ovarian Cancer; Lung Cancer, Colon Cancer; Plasma Cell Neoplasm, Breast Cancer	Recruiting	Sep 30, 2022
NCT04353830	TIGIT	IBI939	270	Ia/Ib	Advanced Malignancies	Recruiting	Dec 31, 2023
NCT04672369	TIGIT	IBI939	36	I	Advanced Lung Cancer	Not yet recruiting	Oct 9, 2024
NCT04672356	TIGIT	IBI939	20	I	Advanced Lung Cancer	Recruiting	Nov 9, 2024
NCT04457778	TIGIT	M6223	35	I	Metastatic Solid Tumors	Recruiting	Sep 14, 2022
NCT04305054	TIGIT	MK-7684	315	I/II	Melanoma	Recruiting	Apr 3, 2030
NCT04305041	TIGIT	MK-7684	200	I/II	Melanoma	Recruiting	Apr 3, 2030
NCT04303169	TIGIT	MK-7684	65	I/II	Melanoma	Recruiting	Apr 3, 2030
NCT04761198	TIGIT	MPH313	125	I/II	Solid Tumor, Adult Advanced Solid Tumor, Metastatic Solid Tumor	Recruiting	Jun 30, 2023
NCT04543617	TIGIT	MTIG7192A	750	III	Esophageal Squamous Cell Carcinoma	Recruiting	Dec 26, 2025
NCT04256421	TIGIT	MTIG7192A	470	III	Small Cell Lung Cancer	Recruiting	Sep 29, 2023
NCT04294810	TIGIT	MTIG7192A	560	III	Non-Small Cell Lung Cancer	Recruiting	Feb 21, 2025
NCT03708224	TIGIT	MTIG7192A	55	II	Cancer, Carcinoma, Squamous Cell Carcinoma, Head and Neck Cancer	Recruiting	Nov 30, 2025
NCT03563716	TIGIT	MTIG7192A	135	II	Non-small Cell Lung Cancer	Active, not recruiting	Oct 30, 2021
NCT03281369	TIGIT	MTIG7192A	410	I/II	Gastric Adenocarcinoma or Gastroesophageal Junction Adenocarcinoma or Esophageal Carcinoma	Recruiting	Feb 11, 2023
NCT03119428	TIGIT	OMP-31 M32	33	I	Locally Advanced Cancer, Metastatic Cancer	Terminated	May 15, 2019
NCT04254107	TIGIT	SGN-TGT	231	I	Non-small Cell Lung Cancer, Gastric Carcinoma, Gastroesophageal Junction Carcinoma, Classical Hodgkin Lymphoma, Diffuse Large B-cell Lymphoma, Peripheral T-cell Lymphoma, Cutaneous Melanoma, Head and Neck Squamous Cell Carcinoma; Bladder Cancer, Ovarian Cancer, Triple Negative Breast Cancer	Recruiting	Mar 31, 2023
NCT04570839	CD112R	COM701	100	I/II	Endometrial Neoplasms, Ovarian Cancer, solid Tumor	Recruiting	Dec, 2023
NCT03667716	CD112R	COM701	140	I	Advanced Cancer, Ovarian Cancer, Breast Cancer, Lung Cancer, Endometrial Cancer, Ovarian Neoplasm, Triple Negative Breast Cancer, Lung Neoplasm, Neoplasm Malignant, Colo-rectal Cancer	Recruiting	Dec, 2021

### Effect of DNAM-1 on NK cells in preclinical trials

DNAM-1 can enhance NK cell cytotoxicity, which is involved in cancer cell recognition, regulation, and death. DNAM-1-induced NK cytotoxicity relies on the interaction of DNAM-1 with its ligands CD155 and CD112, which are highly expressed in cancer cells [51]. In vivo evidence has shown that DNAM-1 controls tumor metastasis, as recently demonstrated in mice lacking DNAM-1 [52, 53]. In coordination with the antitumor response, the overexpression of DNAM-1 ligands (DNAM-1Ls) on the cancer cell surface is induced to identify and eliminate NK cells [26, 54–60]. A previous study showed that antibody-mediated masking of NK cell-activating receptors and costimulatory molecules, such as DNAM-1, natural cytotoxicity receptors (NCRs), and NK cell activating receptor natural-killer group 2, member D (NKG2D), frequently induces melanoma cell lysis after receptor-ligand engagement [61]. As mentioned above, PVR (CD155) and nectin2 (CD112) are ligands for DNAM-1. PVR-expressing neuroblastoma cells are efficiently killed by NK cells when engaging with DNAM-1. Blocking either DNAM-1 or PVR with an anti-DNAM-1 or anti-PVR antibody results in the significant inhibition of NK-mediated tumor cell lysis [26]. Similarly, NK-mediated lysis of tumor cells is enhanced after DNAM-1 (in NK cells) interacts with PVR or Nectin-2 (in target cells), whereas this effect is inhibited by treatment with a mAb targeting DNAM-1 or its receptor [15]. Similarly, soluble DNAM-1 (sDNAM-1) also inhibits the growth of cancer cell lines (K562 and HeLa cells) after binding CD155 or CD112 by enhancing NK cell cytotoxicity, and the inhibitory effect of DNAM-1 is significantly attenuated after DNAM-1 mAb blockade [62]. DNAM-1-mediated NK cell activation is altered by the microRNA miR-30c-1*, which enhances NK cell cytotoxicity in human hepatoma cells by targeting the inhibitory transcription factor HMBOX1 and increases the expression of transmembrane tumor necrosis factor-alpha (mTNF-α) [27]. After treatment with the anti-DNAM-1 mAb LeoA1, miR-30c-1* expression alters NK cell killing capacity [27]. The abovementioned studies demonstrated that anti-DNAM-1 mAbs alter NK cell cytotoxicity and are thus additional novel potential immunotherapeutic agents that inhibit tumors through DNAM-1 agonism. A few studies have demonstrated that DNAM-1 agonists increase DNAM-1 expression on the NK cell surface. Increased DNAM-1 improves NK cell cytotoxicity against melanoma cells and inhibits CNS autoimmunity in experimental autoimmune encephalomyelitis by interacting with the ligand CD155 in dendritic cells [28].

### Effect of DNAM-1 on T cells in preclinical trials

DNAM-1 is expressed on T cells, and several recent preclinical studies have focused on cancer immunotherapy involving targeting DNAM-1 receptors on the T cell surface. DNAM-1 expression affects Treg function, and one study showed that DNAM-1- Tregs are more stable than DNAM-1+ Tregs during in vitro expansion [29]. Blocking the DNAM-1-CD155 interaction promotes Treg expansion and in vitro Treg production. Furthermore, an anti-DNAM-1 mAb prolongs skin allograft survival in a skin allograft model, exhibiting novel therapeutic potential for allogeneic transplantation [29]. In addition to Tregs, DNAM-1-expressing γδ T cells promote cancer cell lysis by engaging ligand nectin-like-5 (CD155) on hepatocellular carcinoma (HCC) cells. Combined mAb-mediated blockade shows that DNAM-1 and NKG2D synergistically affect the cytolytic activity of γδ T cells [30]. The abovementioned studies demonstrated that anti-DNAM-1 mAbs alter T cell cytotoxicity and represent novel potential immunotherapeutic agents against tumors. DNAM-1 + CD8+ tumor-infiltrating T cells possess greater self-renewal ability and cytotoxicity, and DNAM-1 agonist antibody-mediated activation of DNAM-1 augments the effect of TIGIT blockade on CD8 T-cell responses and increases the number of DNAM-1 CD8 T cells, which improves responses to anti-TIGIT therapy for cancer immunotherapy [31].

### Clinical trials

A phase aI/bI clinical trial of the DNAM-1 agonist LY3435151 alone or combined with pembrolizumab (anti-PD-1 mAb) is ongoing in patients with solid tumors, triple-negative breast cancer, gastric adenocarcinoma, head and neck squamous cell carcinoma, cervical carcinoma, high-grade serous ovarian carcinoma, undifferentiated pleomorphic sarcoma, and leiomyosarcoma (NCT04099277).

### TIGIT

T-cell immunoglobulin and immunoreceptor tyrosine-based inhibitory motif (TIGIT) is a member of the immunoglobulin superfamily. It was first identified in 2009 as a coinhibitory receptor on immune cells [63]. TIGIT is a surface protein containing an extracellular IgV-like domain, a type 1 transmembrane domain, and an intracellular domain that contains an ITIM and an ITT-like motif [63]. As highlighted, ITT-like motifs play a crucial role in inhibiting signals. An increasing number of studies have demonstrated that TIGIT is expressed on NK cells, T cells, follicular helper T cells, and follicular regulatory T cells [63–67].

The ligands for TIGIT identified to date include CD112/nectin2 and CD155/PVR, which exhibits the highest affinity. As one of the members of the CD28 family [65], TIGIT shares the ligand CD155 with DNAM-1. The binding of CD155 to TIGIT suppresses T cell activation [35, 63]. In addition to binding to CD155, TIGIT can bind to CD112, and both receptor-ligand interactions inhibit NK cytotoxicity directly through the ITIM motif, which inhibits the NK-mediated killing of tumor cells [64]. CD113 (Nectin-3 or PVRL3) was recently identified as a ligand for TIGIT, which suppresses T cell activity [63]. Nectin4 was originally described as belonging to the nectin family and mediates various cell functions, such as proliferation, differentiation, migration, and invasion [68, 69]. A recent study revealed that Nectin4 is a novel ligand for TIGIT [70]. TIGIT suppresses immune responses mainly through three different mechanisms: direct signaling in cis, induction of ligand signaling in trans, and indirect signaling in competition with costimulatory receptors (Fig. 2).

### Direct signaling in cis

**Fig. 3:**
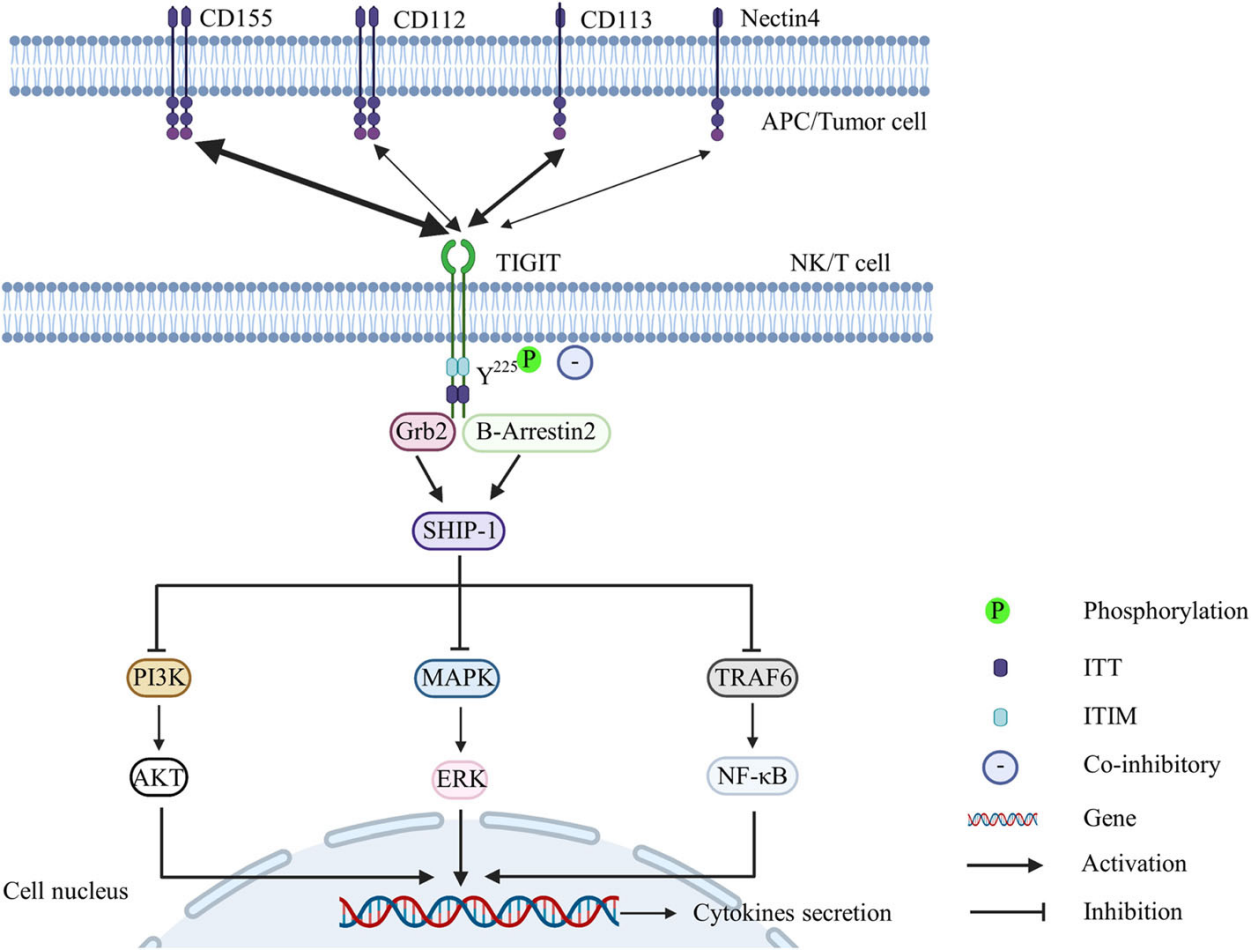
Created with BioRender.com. The bidirectional arrow represents the interaction between TIGIT and its ligands. The thickness of the arrow represents affinity between TIGIT and its common ligands. TIGIT exerts a coinhibitory effect after engagement with its ligands CD155, CD112, CD113, and Nectin4. The phosphorylated Y^225^ of TIGIT binds to cytosolic adapter Grb2 and β-arrestin 2 after engaging with its ligands, leading to recruitment of SHIP1, which eventually influences cytokine secretion by regulating several signaling pathways, such as PI3K, MAPK, TRAF6, and NF-κB

To date, TIGIT direct signaling has been mainly investigated in NK cells (Fig. 3). The phosphorylated Y^225^ residue in the ITT-like motif of TIGIT binds to cytosolic adapter Grb2, which recruits SH2-containing inositol phosphatase-1 (SHIP1), subsequently resulting in the inhibition of the phosphoinositide 3-kinase (PI3K) and mitogen-activated protein kinase (MAPK) signaling pathways [71]. Moreover, the phosphorylation of the two downstream signaling factors AKT and ERK is inhibited, which decreases NK cell cytotoxic activity. In addition, phosphorylated Y^225^ in the ITT-like motif of TIGIT binds to adaptor β-arrestin 2, which is involved in the modulation of immunity through the recruitment of SHIP1. Furthermore, phosphorylated TIGIT inhibits IFN-γ production by impairing tumor necrosis factor (TNF) factor receptor (TNFR)-associated factor 6 (TRAF6) autoubiquitination and nuclear factor kappa B (NF-κB) activation [72]. Similarly, TIGIT exerts immunosuppressive effects on CD8+ T cells by regulating signaling pathways, significantly decreasing the p-AKT/AKT and p-ERK/ERK ratios and increasing p-IκBα levels in HCC [73]. This action eventually results in decreased secretion of interferon-γ (INF-γ), TNF-α, and IL-17A and increased IL-10 secretion [73].

### Induction of ligand signaling in trans

TIGIT exerts an immunosuppressive effect by inducing ligand signaling in trans (Fig. 2). TIGIT binds to its ligands expressed on dendritic cells (DCs). The TIGIT-Fc fusion protein inhibits T cell activation by enhancing the production of IL-10 and diminishing the production of IL-12p40 in DCs, creating an immunosuppressive microenvironment [63]. As mentioned previously, TIGIT is expressed on Treg cells, which are superior in suppressing T cell activation. TIGIT+ Tregs exhibit better suppressive capacity than TIGIT- Tregs because they augment Treg suppression and stability [24, 25], which may be associated with high expression of IL-10, perforin, and TGF-β. Additionally, a study found that deletion of TIGIT in Tregs is effective in inhibiting tumor growth and enhancing CD8+ TIL cytotoxicity [74]. Thus, TIGIT may play a more dominant role in suppressing antitumor immunity via Tregs. However, Foxp3+ Treg cells increase the expression of the effector molecule fibrinogen-like protein 2 (Fgl2), which suppresses proinflammatory T helper 1 (Th1) and Th17 cell responses but not Th2 cell responses [75]. IL-4-expressing Th2 cells promote the differentiation of type 2 tumor-associated macrophages (TAM2), which inhibits T-cell responses by secreting IL-10 and TGF-β and producing arginase-1 and indoleamine 2,3-dioxygenase (IDO) [76]. These cytokines and enzymes consume nutrients within the tumor microenvironment (TME), which regulates T cell activity.

### Indirect competition with costimulatory receptors

PVR-like protein receptors exhibit different affinities for binding to different ligands. TIGIT has the highest affinity for CD155 followed by CD96 and DNAM-1 [63, 77]. Hence, TIGIT exerts an immunosuppressive effect on immune cells by abolishing DNAM-1-mediated costimulation. One study showed that TIGIT indirectly inhibits T cell activation by directly competing with DNAM-1 for binding to CD155 [78]. Additionally, TIGIT can disrupt DNAM-1 cis-homodimerization in human T cells [79]. Both studies suggest that TIGIT inhibits T cell activity by indirectly competing with costimulatory receptors (Fig. 2).

### Preclinical and clinical trials

Evidence from preclinical (Table 1) and clinical trials (Table 2) supports the use of TIGIT-targeted immunotherapies. TIGIT has been shown to be a negative regulator of NK cell or T cell activity in preclinical trials. The number of recruiting, not yet recruiting, active but not recruiting and completed clinical trials assessing the safety and efficacy of a human anti-TIGIT monoclonal antibody for tumor treatment continues to increase.

### Effect of TIGIT on NK cells in preclinical trials

Given the cytotoxicity of NK cells, increasing evidence has shown that TIGIT+ NK cell cytotoxicity is inhibited and that this inhibition is accompanied by decreased cytokine production. The cytotoxicity of human YTS NK cells transfected with TIGIT is inhibited [64]. The redirected killing ability of both human [64] and mouse primary NK cells [80] is decreased after TIGIT engages with its ligand CD155. TIGIT expression levels are related to the NK cell phenotype and functional heterogeneity [81]. Compared to NK cells with high expression of TIGIT, NK cells with low TIGIT expression exhibit an increased cytokine secretion capacity, degranulation activity and cytotoxicity [81]. In addition, MDSC-induced NK cell inhibition is associated with high expression of TIGIT and the TIGIT/CD155 interaction [82]. These data consistently show that high expression of the PVR receptor TIGIT exerts a coinhibitory effect on NK cells. Therefore, blockade of TIGIT or the TIGIT-ligand interaction represents a potentially promising cancer therapy. Studies have shown that blockade of TIGIT restores NK cell exhaustion and promotes NK cell-dependent tumor immunity, enhancing degranulation and IFN-γ production in healthy donor CD56dim NK cells. This phenomenon is more apparent in combination with other checkpoint receptors [32, 33].

### Effect of TIGIT on T cells in preclinical trials

Aberrant TIGIT expression results in tumor immune escape in the TME. Several groups have consistently reported that TIGIT is highly expressed on CD8+ TILs in many cancers, such as nonsmall cell lung cancer, colon cancer, melanoma [34, 79], and acute myelogenous leukemia [83]. Both melanoma and AML patients have an immunosuppressed microenvironment, largely due to low production of cytokines caused by high TIGIT expression [34, 83]. TIGIT expression is upregulated and DNAM-1 expression in CD8+ TILs is decreased in melanoma and AML patients, indicating the role of a TIGIT/DNAM-1 imbalance in tumor progression. These data consistently demonstrate that TIGIT negatively regulates T-cell function. Knockdown of TIGIT in CD8+ T cells from AML patients reverses cytotoxic and proliferative capacity defects [83]. Moreover, blockade of TIGIT or an anti-TIGIT antibody enhances T cell activation and restores CD8+ T exhaustion, enhancing antitumor activity in gastric cancer, colon cancer, myeloma, and head and neck squamous cell carcinoma [34–40]. In addition, TIGIT regulates Treg function. As mentioned above, Tregs are superior in suppressing T cell activation, and anti-TIGIT treatment reduces the proportion of CD4+ Tregs and inhibits the suppressive ability of Tregs [39–42], suggesting another pathway for cancer immunotherapy.

### Clinical trials

Evidence from clinical trials supports the use of TIGIT-mediated immunotherapies. To date, twelve human anti-TIGIT monoclonal antibodies have been developed, i.e., AB154 (NCT04656535, NCT03628677, NCT04262856, NCT04736173), ASP8374 (NCT03945253, NCT03260322), BGB-A1217 (NCT04693234, NCT04732494, NCT04047862, NCT04746924), BMS-986207 (NCT04150965), COM902 (NCT04354246), IBI939 (NCT04353830, NCT04672369, NCT04672356), M6223 (NCT04457778), MK-7684 (NCT04305054, NCT04305041, NCT04303169), MPH313 (NCT04761198), MTIG7192A (NCT04543617, NCT04256421, NCT04294810, NCT03708224, NCT03563716, NCT03281369), OMP-31 M32 (NCT03119428), and SGN-TGT (NCT04254107). The safety and efficacy of these antibodies alone or combined with other immune checkpoint inhibitors, such as anti-PD-1, anti-PD-L1, anti-CTLA-4, anti-A2aR and anti-A2bR, are being studied in twenty-eight ongoing clinical trials in patients with human tumors; these trials are described in Table 2.

### CD96

CD96, which was identified as TACTILE (T cell activation, increased late expression), is also a member of the Ig superfamily [84]. Human CD96 comprises one single-pass transmembrane region, three extracellular Ig-like domains/loops, and one cytoplasmic domain. These structural characteristics of CD96 enhance the complexity of its ligand interactions. Short cytoplasmic domains contain multiple binding sites with signal transduction capabilities, but the characteristics of intracellular signal transduction are less understood. The cytoplasmic domain of CD96 in both mice and humans contains a single putative ITIM motif, which is associated with its inhibitory function [85]. Moreover, the human CD96 cytoplasmic domain consists of a YXXM motif [86], which may activate receptors in certain contexts.

CD96 is expressed primarily on conventional αβ and γδ T cells, NK cells, and a proportion of hematopoietic stem cells in humans [84, 87]. In mice, CD96 is expressed on αβ and γδ T cells, NK cells, and NKT cells [52, 88]. However, CD96 is not expressed on B cells, DCs, resident or inflammatory monocytes, neutrophils, or granulocytes [52, 88–90]. Two ligands of CD96 have been identified: CD155 and CD111 (nectin-1). Similar to TIGIT and DNAM-1, CD96 interacts with CD155 to regulate NK cell and T cell functions. CD96 also interacts with CD111 (nectin-1) to regulate NK cell and T cell functions [88, 89, 91]. The costimulatory and coinhibitory effects of NK cells and T cells after CD96 engages with its ligands are complex and are described in the following section.

### CD96 signaling pathway

**Fig. 4:**
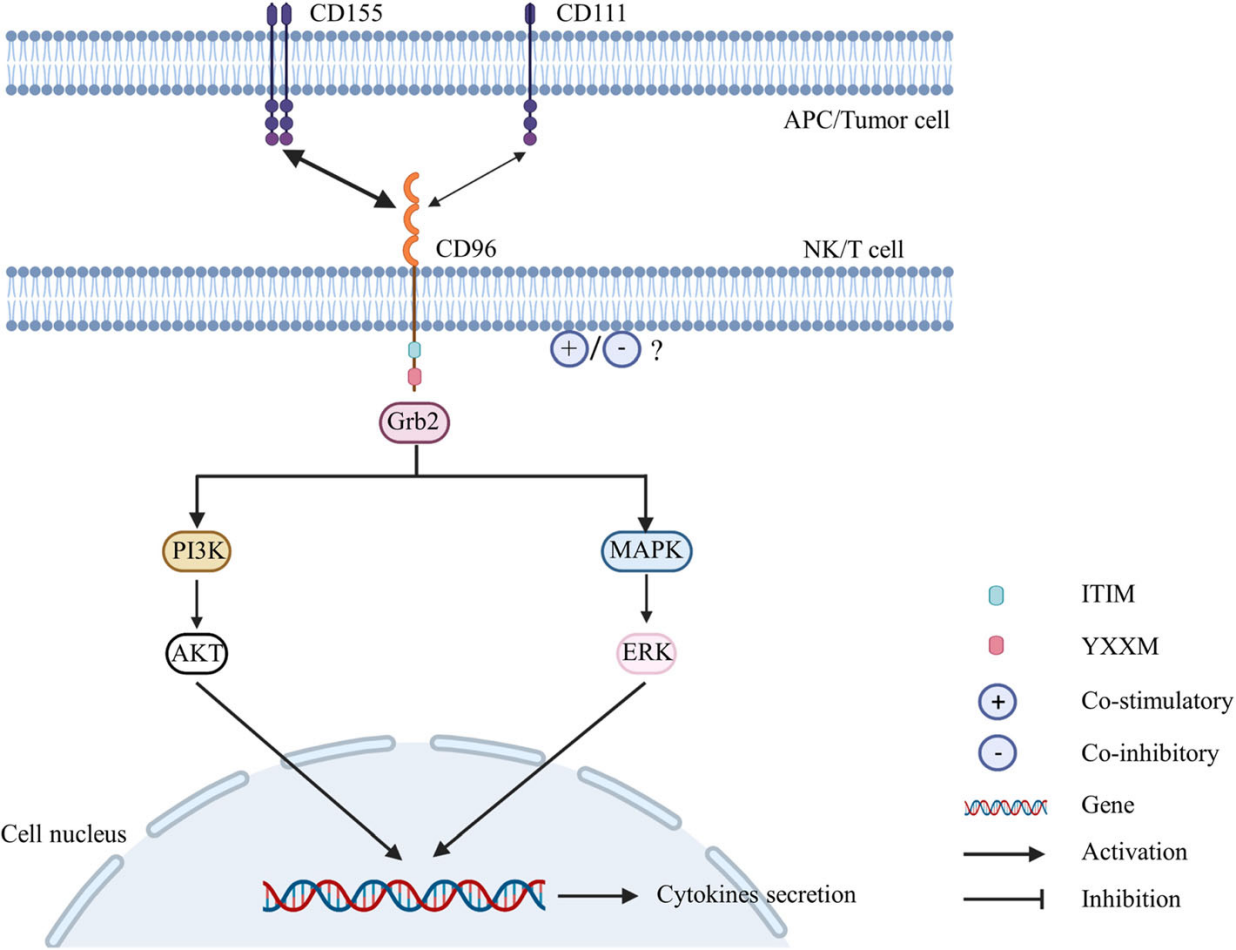
Created with BioRender.com. The bidirectional arrow represents the interaction between CD96 and its ligands. The thickness of the arrow represents affinity between CD96 and its common ligands. CD96 engages with its ligands CD115 and CD111 and exerts costimulatory effects in T cells by regulating the ERK and PI3K signaling pathways. However, studies have investigated whether CD96 can exert coinhibitory effects in NK cells and T cells, but the molecular changes are unknown and require further research

CD96 regulates T cell function by inducing signaling pathways (Fig. 4). A region of the mouse CD96 intercellular domain binds with Grb2 and the SH2 and SH3 domains, which transduce signals [92, 93]. The mechanisms underlying the regulation of T cells by the ERK signaling pathway have been investigated [94]. Beads coated with CD3/CD96 induce marked ERK phosphorylation. ERK is rapidly and transiently phosphorylated, and a dramatic reduction in signaling and differentiation is subsequently observed [46]. This finding indicates that CD96 functions as a costimulatory receptor in mouse CD8+ T cells by regulating the ERK signaling pathway. Human CD96 is similar to mouse CD96, and it is worth noting that human CD96 contains a YXXM motif. CD3/CD96 stimulation also enhances the phosphorylation of ERK in human CD8+ T cells [46]. Additionally, the YXXM motif binds the p85 subunit of PI3K [95], which phosphorylates and activates the downstream effector AKT. These data demonstrate that CD96 functions as a costimulatory receptor in human CD8+ T cells by regulating the PI3K/AKT signaling pathway [46].

### Preclinical and clinical trials of CD96-targeted therapies

Current preclinical data reveal that CD96 may have a coactivating or coinhibitory effect on human and/or mouse NK cells or T cells (Table 1) as described below. This apparent difference can be explained by the inherent differences in CD96 signaling between mice and humans as described previously; however, this effect has not been confirmed to date. Alternatively, these inconsistent results have been confirmed by several studies in mice and humans.

### Effect of CD96 on NK cells in preclinical trials

CD96 negatively regulates human and mouse NK cell activity. CD96 attenuates NK cell cytotoxicity by competing with DNAM-1 for CD155 binding [52]. CD96(−/−) mice display hyperinflammatory responses to the bacterial product lipopolysaccharide (LPS) and show resistance to carcinogenesis and experimental lung metastases. These results indicate that CD96 negatively controls cytokine responses by NK cells [52]. Hence, blocking CD96 is a potential cancer immunotherapy. An increasing number of preclinical trials have confirmed this hypothesis. Blocking CD96 or administering an anti-CD96 antibody alone enhances NK cell function, which significantly reduces experimental and spontaneous lung metastases in mice. This effect is more apparent when these strategies are used in combination with anti-CTLA4 antibodies, anti-PD-1/PD-L1 antibodies, and doxorubicin for cancer immunotherapy [43, 96]. In human HCC samples, the number of CD96+ NK cells is significantly increased, and these cells are functionally exhausted and exhibit impaired IFN-γ and TNF-α production, high gene expression of IL-10 and TGF-β1, and low gene expression of T-bet, IL-15, perforin, and granzyme B [44]. In contrast, human NK cell cytotoxicity is restored and enhanced in the presence of an anti-CD96 antibody that blocks CD96 and its interaction with CD155 [44, 89]. Two anti-CD96 antibodies block the CD96-CD155 interaction (3.3 and 6A6), and one antibody (8B10) does not. Consistent with its inability to block CD96-CD155 interactions, 8B10 retains antitumor activity in CD155-deficient mice, whereas 3.3 and 6A6 lose potency in CD155-deficient mice, suggesting that CD96-targeted antibodies promote NK cell antitumor activity without blocking the CD96-CD155 interaction [45].

### Effect of CD96 on T cells in preclinical trials

CD96 regulates T cell activity rather than NK cell activity. However, the potential coactivating and coinhibitory effects of CD96 on human and/or mouse T cells are complicated. CD96 promotes human CD8+ T cell cytotoxic activity through the MEK-ERK pathway. Therefore, the absence of CD96 or Ab-mediated CD96 blockade on CD8+ T cells abolishes IFN-γ and/or TNF-α production, which is associated with CD8+ T cell activation in in vivo models [46]. However, CD96−/−CD8+ T cells in mice promote tumor growth more than CD96-sufficient CD8+ T cells. In addition, anti-CD96 therapy is effective in enhancing CD8+ T activity and limiting tumor growth and is more effective when administered in combination with blockade of a number of immune checkpoints, including PD-1, PD-L1, TIGIT, and CTLA-4 [47]. CD96low Th9 cells in (Rag1−/−) mice can cause severe weight loss, intestinal and colonic inflammation, and destruction of allogeneic skin grafts, showing expansion and tissue accumulation. In contrast, CD96high Th9 cells do not cause colitis and exhibit reduced expansion and migratory potential [48]. Interestingly, blockade of CD96 significantly restores the expansion and inflammatory properties of CD96high Th9 cells [48]. These results that CD96 plays an inhibitory role in suppressing IL-9-expressing Th9 cell activity, providing novel opportunities for the treatment of IL-9-associated inflammation, such as inflammatory bowel disease (IBD).

### Clinical trials

Although the abovementioned preclinical trials have suggested that CD96 on NK cells and T cells may be potential targets for cancer immunotherapy, the expression levels of this immune checkpoint receptor differ on mouse and/or NK cells and T cells. In addition, CD96-associated studies and clinical trials in human cancer patients are lacking. Thus, further studies of the potential of CD96 as a target molecule for cancer immunotherapy are needed.

### CD112R

CD112R, previously named poliovirus receptor-related immunoglobulin domain-containing protein (PVRIG), is a novel member of the PVR-like cosignaling network. CD112R is a 36-kD transmembrane monomer composed of a single extracellular IgV domain, one transmembrane domain, and a long intracellular domain [97]. The human CD112R intracellular domain comprises two tyrosine residues, one of which is part of an ITIM-like motif and a potential site for phosphatases [98]. The extracellular domain sequences of human and mouse CD112R exhibit ∼65.3% homology. In addition, phylogenetic tree analysis of the first IgV of the PVR family revealed that CD112R is closely related to PVR-like proteins [97].

CD112R is expressed at low and variable levels on the surface of freshly isolated T-cells and NK cells (predominantly on CD8+ T-cells, which are mainly memory/effector but not naïve cells) and on both CD16+ and CD16- NK cells in humans. CD112R is not detected in B-cells (CD19+), naïve (CD45RA + CCR7+) or helper (CD4+) T-cells, monocytes (CD14+), or neutrophils (CD66b+) at the protein level. Moreover, CD112R is not detected on DCs in humans. CD112R expression on the surface of T cells is further upregulated by treatment with anti-CD3 and anti-CD28 antibodies [97]. CD112R strongly interacts with CD112/Nectin2 and acts as a coinhibitory receptor that suppresses receptor-mediated signals and inhibits immune cell proliferation. CD112R and DNAM-1 share a common binding site on CD112. CD112R competes with DNAM-1 for binding to CD112 on T cells and mediates an inhibitory signal. CD112R is the receptor for CD112 with the highest affinity in both humans and mice and mediates the interaction of immune cells with DCs and tumor cells [97].

### CD112R signaling pathway

**Fig. 5:**
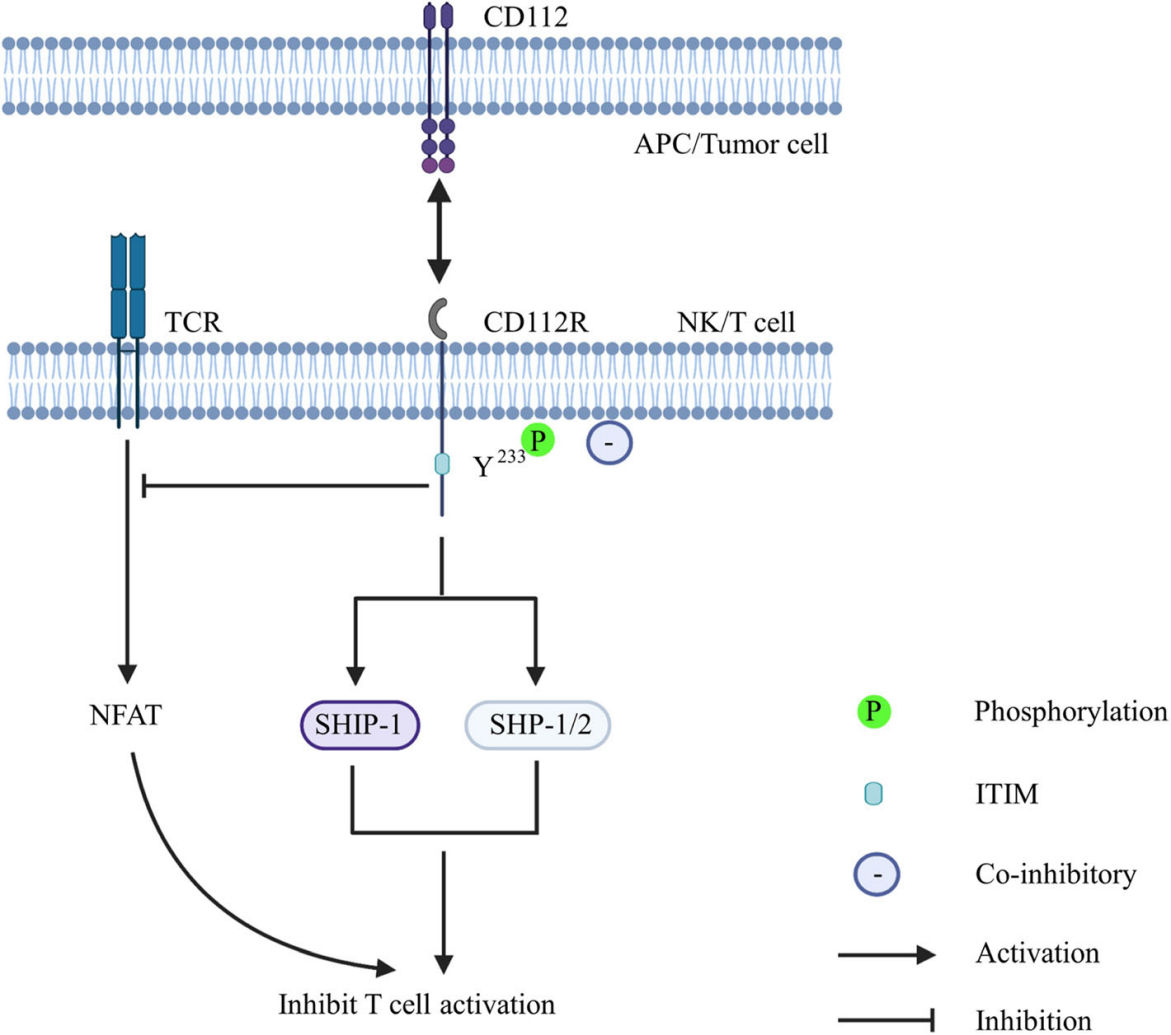
Created with BioRender.com. The bidirectional arrow represents the interaction between CD112R and its ligand. CD112R engages with its ligand, and the phosphorylated Y^233^ residue of the ITIM-like motif recruits SHIP-1 and SHP1/2 to mediate signal transduction, leading to a decrease in T cell cytotoxicity. Additionally, CD112R potentially represents a new coinhibitory receptor that suppresses T cell receptor-mediated NFAT activation

The Y^233^ residue of the ITIM-like motif in the CD112R intracellular domain is phosphorylated by tyrosine phosphatases to mediate signal transduction [97] (Fig. 5). This finding was confirmed in the CD112Rhigh leukemia T cell line Molt4. The results showed that SHIP is strongly associated with CD112R in untreated Molt4 cells, and this interaction further increases upon pervanadate treatment. In addition, SHP-1 and SHP-2 are weakly associated with CD112R in untreated Molt4 cells, but pervanadate treatment further enhances these associations [97]. All these results suggest that CD112R transduces signals by recruiting tyrosine phosphatases. Additionally, this study suggested that CD112R potentially represents a new coinhibitory receptor that suppresses T cell receptor-mediated NFAT activation.

### Effect of CD112R on NK cells in preclinical trials

Few studies on cancer immunotherapies have focused on targeting CD112R PVR-like checkpoint proteins. CD112R is expressed on human NK cells, although its function in this cell type is unclear. The number of human NK cells expressing the PVR receptor CD112R or TIGIT (CD56+) decreases with decreased IFN-γ production in CD112-positive breast cancer, indicating that CD112R engagement with the ligand CD112 suppresses NK cell cytotoxicity [49]. Xu and coworkers showed that blockade of TIGIT and CD112R separately or together improves the effect of trastuzumab on breast cancer by enhancing NK cell activity [49].

### Effect of CD112R on T cells in preclinical trials

Interestingly, instead of mediating NK cells, CD112R plays a vital role in T-cell-mediated cancer immunity. CD112R is a distinct inhibitory signaling molecule on human CD8+ T cells that decreases IFN-γ production [50]. COM701 is a humanized anti-CD112R hinge-stabilized IgG4 that binds to human CD112R and disrupts the CD112R–CD112 interaction, which enhances T-cell function. This effect is enhanced by TIGIT or PD-1 blockade in Mel-624 cells and Panc.05.04 cells [50]. Furthermore, COM701 + nivolumab (anti-PD-1) or COM701 alone result in better outcomes in patients with advanced solid tumors in a phase I clinical trial [50]. CD112R is a novel member of the PVR-like protein cosignaling network. Research on the interactions of CD112R with other poliovirus-like ligands is lacking, and these interactions need to be studied further.

### Clinical trials

One human anti-CD112R monoclonal antibody, COM701, has entered clinical trials. When combined with blockade of CD112R and TIGIT, PD-1 exerts a more powerful antitumor effect in preclinical models. Thus, two clinical trials are being performed to evaluate the safety and efficacy of combination anti-TIGIT (BMS-986207) and anti-PD-1 (Opdivo, Nivolumab) therapy as well as anti-CD112R monotherapy. These trials are ongoing in patients with endometrial neoplasms, ovarian cancer, solid tumors (NCT04570839), advanced cancer, ovarian cancer, breast cancer, lung cancer, endometrial cancer, ovarian neoplasm, triple-negative breast cancer, and colorectal cancer (NCT03667716).

## Conclusion

In recent years, immunotherapy has represented one of the most promising therapeutic methods for cancer therapy and has attracted a considerable amount of attention. Immunotherapy utilizes the body’s immune system to kill and eliminate infected and transformed cells by enhancing NK and T cell activities and is safe and highly effective. Although the costimulatory and coinhibitory mechanisms of NK and T cells after receptors in the PVR-like protein cosignaling network engage with their ligand have been studied, the detailed intracellular signaling mechanisms are unclear and need to be elucidated. Recently, anti-PD-1 mAbs were approved by the Food and Drug Administration (FDA) for the treatment of many cancers. The challenge in the future will be to develop mAbs targeting molecules in this cosignaling network to treat cancers. Based on the abovementioned data, we know that the efficacy of strategies targeting these cosignaling network receptors is increased when used in combination with other existing immune checkpoint blockade therapies, such as anti-CTLA-4 or anti-PD-1 mAbs, to treat cancer. The next challenge we face is the development of combination therapies involving mAbs targeting molecules in this novel cosignaling network and other unknown immune checkpoints for cancer treatment. More importantly, clinical results indicate that many cancers, such as kidney, gallbladder, or bile duct malignant tumors, do not respond or respond poorly to immune checkpoint blockade therapy. How numerous innate receptors regulate NK cell and T cell responsiveness spatially and temporally is unclear and therefore should be investigated to identify other receptors and their ligands that share the same structure.

## Data Availability

Not applicable.

## Abbreviations

PVR: Poliovirus receptor
DNAM-1: DNAX accessory molecule-1
TIGIT: T-cell immunoglobulin and immunoreceptor tyrosine-based inhibitory motif
NK: Natural killer
CTLA-4: Cytotoxic T lymphocyte antigen-4
PD-1: Programmed cell death 1
PD-L1: Programmed cell death-ligand 1
Ig: Immunoglobulin
ITIM: Immunoreceptor tyrosine-based inhibitory motif
IgV: Ig variable
mAb: Monoclonal antibody
CNS: Central nervous system
HCC: Hepatocellular carcinoma
ITT: Ig tail-tyrosine
PKC: Protein kinase C
LFA-1: Leucocyte function-associated antigen-1
SLP-76: SH2 domain-containing leukocyte phosphoprotein of 76 kDa
Grb2: Growth factor receptor-bound protein 2
PLC- γ2: phospholipase Cγ2
PLC- γ1: phospholipase Cγ1
PI3K: Phosphatidylinositol 3’kinase
DNAM-1Ls: DNAM-1 ligands
NKG2D: NK cell activating receptor natural-killer group 2, member D
SHIP1: SH2-containing inositol phosphatase-1
MAPK: Mitogen-activated protein kinase
INF-γ: Interferon-γ
TNF-α: Tumor necrosis factor-alpha
TGF-β: Transforming growth factor-beta
